# Efficacy and safety of PEG-rhG-CSF versus rhG-CSF in preventing chemotherapy-induced-neutropenia in early-stage breast cancer patients

**DOI:** 10.1186/s12885-023-11198-2

**Published:** 2023-07-26

**Authors:** Yantao Jiang, Ju Zhang, Jianxin Zhong, Hao Liao, Jiayang Zhang, Yaxin Liu, Yuehua Liang, Huiping Li

**Affiliations:** 1 Department of Breast Oncology, Key Laboratory of Carcinogenesis and Translational Research (Ministry of Education/Beijing), Peking University Cancer Hospital & Institute, No. 52, Fucheng Road, Haidian District, Beijing, 100142 China; 2 Department of Nuclear Medicine, the Affiliated Hospital of Qingdao University, Qingdao, China

**Keywords:** PEG-rhG-CSF, rhG-CSF, Breast cancer, Chemotherapy-induced- neutropenia, Febrile neutropenia

## Abstract

**Background:**

To compare the clinical value of recombinant human granulocyte colony-stimulating factor (rhG-CSF) and pegylated rhG-CSF(PEG-rhG-CSF) in early-stage breast cancer (EBC) patients receiving adjuvant chemotherapy, compare the efficacy of PEG-rhG-CSF with different dose and explore the timing of rhG-CSF rescue treatment.

**Methods:**

Patients in two PEG-rhG-CSF subgroups were given 3 mg or 6 mg PEG-rhG-CSF within 24 ~ 48 h after chemotherapy for preventing myelosuppression, while patients in the rhG-CSF group were given rhG-CSF. Observation indicators include the incidence of febrile neutropenia (FN) and grade 3/4 chemotherapy-induced-neutropenia (CIN), the overall levels and nadir values of white blood cells (WBC) and absolute neutrophil count (ANC), comparison of WBC and ANC curves over time, the incidence of CIN-related complications, the incidence of adverse events in each group and the timing of rescue treatment for rhG-CSF.

**Results:**

There was no significant difference in the incidence of FN in the first cycle among the groups (*P* = 0.203). But the incidence of ≥ 3 grade CIN in two PEG-rhG-CSF subgroups was significantly lower than that in the rhG-CSF group (*P* < 0.001). The overall WBC and ANC levels in the PEG-rhG-CSF group were significantly higher than those in the rhG-CSF group (*P* < 0.001). In terms of CIN-related complications, less chemotherapy delay rate (1.1 vs. 7.5%, *P* = 0.092), less dose reduction rate (6.9 vs. 7.5%, *P* = 1.000), less antibiotic use rate (3.4 vs. 17.5%, *P* = 0.011) and less proportion of rhG-CSF rescue therapy (24.1 vs. 85.0%, *P* < 0.001) in the PEG-rhG-CSF group, and there were no significant differences between PEG-rhG-CSF subgroups. In the incidence of adverse events among the groups, there were no statistical differences. All patients undergoing rhG-CSF rescue treatment were mainly 4 grade (63.6%) and 3 grade (25.5%) CIN, and 10.9% of patients with 1 ~ 2 grade CIN who had high infection risk or had been infected.

**Conclusion:**

PEG-rhG-CSF has better efficacy and equal tolerance compared with rhG-CSF in preventing CIN in EBC patients receiving EC regimen. Moreover, a half-dose 3 mg PEG-rhG-CSF also had good efficacy. Last, patients with ≥ 3 grade CIN and others who have been assessed to be at high risk of infection or have co-infection should consider rhG-CSF or even antibiotic rescue treatment.

## Background

Breast cancer is the most common malignant tumor in women, posing a serious threat to women's health, and has surpassed lung cancer as the world's most prevalent cancer [1, 2]. Despite recent advances in the treatment of breast cancer with surgery, targeted therapy, and immunotherapy, chemotherapy still plays an unshakable role as the cornerstone of systemic therapy [3]. According to the International Breast Cancer Study Group, chemotherapy can reduce the risk of death by 30% and the risk of recurrence by 28% in patients with EBC [4]. However, chemotherapy is a systemic treatment with poor drug selectivity, and it kills many normal cells while attacking tumor cells, which damages human immune function. In particular, the two-drug or three-drug chemotherapy regimens consisting of anthracyclines, paclitaxel, platinum, cyclophosphamide, and other chemotherapeutic agents commonly used in breast cancer chemotherapy are classified by guidelines as medium–high risk regimens for FN (risk of FN ≥ 10%) [5]. Severe CIN, especially FN, is extremely dangerous, as it can not only cause immune deficiency and thus induce infection but also cause reduction of the established chemotherapy dose, delay of chemotherapy or even chemotherapy shedding, which may reduce the patient's treatment effect and affect the prognosis [6, 7]. In addition, the re-hospitalization rate of patients due to FN and other infections increases, and the costs associated with antibiotics and antipyretics also increase, making the clinical and financial burden an important factor affecting the quality of life of oncology patients. Therefore, the above-mentioned problems have attracted more and more attention from clinicians in breast cancer chemotherapy. G-CSF, a cytokine that can stimulate the proliferation and differentiation of neutrophil precursors and enhance the function of mature neutrophils, has been widely used in clinical practice because it can prevent and treat CIN and FN and reduce the hematological toxicity of chemotherapy [3, 8]. G-CSF is mainly divided into two types: PEG-rhG-CSF and rhG-CSF.

RhG-CSF is a short-acting form of G-CSF, which was first approved for marketing in the United States in 1991 [9]. The emergence of rhG-CSF has greatly reduced the incidence and severity of CIN and FN. However, rhG-CSF is mainly metabolized by the kidneys and has a short half-life, which requires repeated daily injections and close monitoring of blood count, which not only increases the risk of infection at the injection site, but also reduces patients' quality of life and compliance with the drug [10]. PEG-rhG-CSF, which was approved by the FDA in 2002, is a protein covalently bound to polyethylene glycol (PEG) at the N-terminal end of the rhG-CSF and it is a long-acting form of G-CSF [11]. PEG-rhG-CSF not only has a large molecular weight, but also changes the method of drug clearance [12]. Compared with rhG-CSF, its half-life is prolonged, concentration stability is enhanced, immunogenicity and antigenicity are reduced, and only one dose per cycle is required to obtain at least non-inferior efficacy and safety compared with frequent injections of rhG-CSF, reducing the number of hospital visits and the risk of cross-infection. It is a breakthrough in the history of adjuvant chemotherapy drug administration, which greatly improves the convenience of drug administration and patient compliance [13–15].

However, the affinity between rhG-CSF and the receptor may be reduced after polyethylene glycosylation modification. Since PEG-rhG-CSF was launched, many clinical studies comparing the efficacy and safety of the two agents of G-CSF have been conducted. A systematic review containing 41 clinical studies involving multiple tumor types including breast cancer, non-small cell lung cancer, and non-Hodgkin's lymphoma noted that PEG-rhG-CSF was superior to rhG-CSF in reducing CIN, FN, readmission rate, and antibiotic use, with statistically significant differences [16]. However, a clinical study including 5 Meta-analyses and involving 9562 patients of multiple tumor types showed that the incidence of FN in the PEG-rhG-CSF group was lower than that in the rhG-CSF group, but the difference was Statistical findings were unstable [17]. Some clinical studies concluded that there was no significant difference in the efficacy and safety of the two groups in the prevention of CIN and FN [18, 19]. In conclusion, the efficacy and safety of PEG-rhG-CSF versus rhG-CSF need to be further investigated, especially in breast cancer patients, and several Meta-analyses have shown that there is no consensus on whether PEG-rhG-CSF is superior to rhG-CSF [13, 20]. In addition, due to the short period of time since the PEG-rhG-CSF has been on the market in China, there are few real-world studies, especially those using a unified treatment regimen to compare PEG-rhG-CSF and rhG-CSF. Therefore, we still need more evidence-based medical evidence.

Chinese Society of Clinical Oncology (CSCO), National Comprehensive Cancer Network (NCCN), American Society of Clinical Oncology (ASCO) guidelines recommend that PEG-rhG-CSF should be administered individually at 100 ug/kg based on body weight or a fixed dose of 6 mg [5, 21]. The fixed dose is widely used in clinical practice because it is convenient to administer. It has been shown that the pharmacokinetics of PEG-rhG-CSF is non-linearly correlated with the dose, and its clearance is lower at higher doses. In addition, body weight is an important factor in drug clearance, with higher clearance in those with higher body weight [15]. Therefore, it is worth considering whether fixed dose administration will reduce the efficacy in large weight groups and whether it will aggravate the adverse drug reactions due to slow clearance in small weight groups. Cao W et al. used 4.5 mg PEG-rhG-CSF prophylactically and also obtained significant benefits in patients treated with ddEC regimen for breast cancer, with lower incidence of FN and severe CIN, delayed chemotherapy, bone pain and other adverse effects [22]. In addition, Zhou JH et al. compared the efficacy and safety of two fixed doses of PEG-rhG-CSF, 3 mg and 6 mg, in the prevention of myelosuppression in elderly patients with gastrointestinal tract tumors receiving chemotherapy, and showed that 3 mg half dose was effective, with no significant difference in the incidence of FN (7.9 vs. 11.1%, *P* > 0.05) and the duration of CIN between the two groups [23]. Although the sample size of this study was small (47 cases) and the results may be biased, it still gives us some indication.

In clinical practice, we also found that even if PEG-rhG-CSF is used, its dosage is not uniform. Moreover, the comparative efficacy of half-dose and full-dose Peg-rhG-CSF administration in breast cancer is rarely reported in the literature. Therefore a study based on real world data is urgently needed to elaborate these problems in practical work, and our study is based on this purpose.

Prophylactic application of PEG-rhG-CSF and rhG-CSF can’t completely avoid the hematologic toxicity of chemotherapy. The results of several real-world studies have shown that a certain percentage of severe CIN and FN requiring rhG-CSF rescue therapy after prophylactic application of both drugs still occur, which may cause serious infections, increase rehospitalization rates and even endanger lives if not properly managed [24]. Therefore, it is very important to strictly grasp the timing of rhG-CSF rescue therapy. In clinical practice, the application of rhG-CSF still has many irrational phenomena, especially in rescue treatment. The above problems were also fully illustrated in the study of Wang YH et al. [25]. In this study, 243 breast cancer patients were included, of which 74 were treated with rhG-CSF, 31.08% (23/74) of which were irrationally treated, including improper timing (5/23), insufficient duration (9/23) and inappropriate indications (9/23). In addition, the CSCO guidelines suggest that patients with prophylactic PEG-rhG-CSF should strictly refer to the dosing indications for rhG-CSF rescue therapy [5]. In China, there are few reports of real-world studies to explore the timing of subsequent rhG-CSF rescue therapy for breast cancer patients with prophylactic PEG-rhG-CSF.

Based on the above research background, this study was conducted to retrospectively analyze EBC patients who were treated with EC regimen chemotherapy in the real world, to compare the efficacy and safety of PEG-rhG-CSF and rhG-CSF in preventing CIN, FN, and to compare the efficacy between 3 and 6 mg subgroups of PEG-rhG-CSF, and to investigate the timing of rhG-CSF rescue therapy. To provide more evidence-based medical evidence for the clinical value of both long- and short-acting doses of G-CSF, to guide clinical application, and also to provide new ideas for the dose selection of PEG-rhG-CSF in practical application, enhance the objective understanding of clinicians about the two drugs, and promote the rational application and standardized treatment of the drugs.

## Materials and methods

### Patients

From June 2014 to June 2021, 127 female EBC patients were selected from the Department of Breast Cancer Medicine, Peking University Cancer Hospital. (1) Inclusion criteria: ① female patients with breast cancer diagnosed by pathological histology; ② TNM stage I-III (AJCC 8th edition); ③ age 18–70 years (including both ends); ④ at least 2 cycles postoperative adjuvant chemotherapy with EC regimen; ⑤ Eastern Cooperative Oncology Group (ECOG) score ≤ 1; ⑥ no serious cardiac function and liver and kidney dysfunction, all indicators are below 2.5 times the normal value; ⑦ normal bone marrow hematopoietic function, all indicators at baseline before chemotherapy are normal; ⑧ no bleeding tendency. (2) Exclusion criteria: ① advanced or inflammatory breast cancer; ② combination of other malignant tumors under treatment; ③ combination of bone marrow hematopoietic dysfunction or other serious comorbidities; ④ previous bone marrow transplantation or hematopoietic stem cell transplantation; ⑤ presence of uncontrollable infection; ⑥ recent use of drugs affecting white blood cells; ⑦ allergy to biological products such as G-CSF; ⑧ presence of other personal reasons for not being able to cooperate.

### Treatment

All patients were treated with postoperative adjuvant chemotherapy using the EC regimen at the following doses and methods: epirubicin 90 mg/m^2^ d1-2 and cyclophosphamide 600 mg/m^2^ d1 for 21 d. Patients in two PEG-rhG-CSF subgroups were prophylactically applied PEG-rhG-CSF 6 mg or 3 mg subcutaneously once 24–48 h after the end of chemotherapy, and patients in the rhG-CSF group were prophylactically applied rhG-CSF 5ug/kg/d subcutaneously once 24–48 h after the end of chemotherapy for 3–5 d. And the patients who need rhG-CSF rescue therapy received daily subcutaneous injections of rhG-CSF at a dose of 5 μg/kg/d until ANC returned to normal or nearly normal (when ANC rose above 2 × 10^9^/L).

### Observation items

The first and last routine blood monitoring was performed within 3 days before and around day 21 after chemotherapy, respectively, and at least one more routine blood test was required during the treatment cycle. All routine blood results were collected and time-stamped, with emphasis on WBC, ANC, Hemoglobin (HGB) and Platelet (PLT) changes. In addition, the highest body temperature per day during the treatment cycle was recorded for each patient, and if the temperature was ≥ 38 °C, the temperature was monitored continuously every 1 h.

### Efficacy and safety measurements

(1) Primary study endpoints: Cycle 1 FN incidence (specifically, a severe decrease in ANC i.e. ANC < 0.5 × 10^9^/L or < 1.0 × 10^9^/L but expected to decrease to less than 0.5 × 10^9^/L after the following 48 h, in combination with fever i.e. oral temperature ≥ 38.3 °C or > 38.0 °C for more than 1 consecutive hour). (2) Secondary research endpoints: ① the incidence of ≥ 3 grade CIN (defined as ANC < 1.0 × 10^9^/L); ② WBC and ANC level distribution and ANC nadir in each group; ③ comparison of WBC and ANC change curves over time; ④ incidence of CIN-related complications: chemotherapy delay rate (defined as chemotherapy delayed ≥ 3 days from the scheduled chemotherapy due to CIN), dose reduction rate (defined as chemotherapy dose reduction ≥ 15% in the next cycle due to CIN), antibiotic use rate and rhG-CSF rescue treatment rate; ⑤ comparison of adverse effects between different types and doses of G-CSF(Safety was assessed by the incidence of adverse events using preferred terms designated by the NCI Common Terminology Criteria for Adverse Events (CTCAE version 5.0); ⑥ timing of rhG-CSF rescue treatment.

## Statistical analysis

Data were summarized and analyzed by SPSS 26.0 software, and graphs were produced by GraphPad Prism 9.0 and Excel software. All data were tested for normality, and quantitative data conforming to normal distribution were compared between two groups using t-test and among three groups using ANOVA, and the above results were expressed as mean ± standard deviation; qualitative data were analyzed by chi-square test or Fisher's exact test, and data were expressed as frequencies (percentages). The nonparametric rank sum test was used for data that did not conform to a normal distribution. The significance level of all statistical tests was set at 0.05, and the two-test confidence intervals (CIs) were set at 95%, with *P* < 0.05 representing statistical significance.

## Results

### Patients

**Fig. 1:**
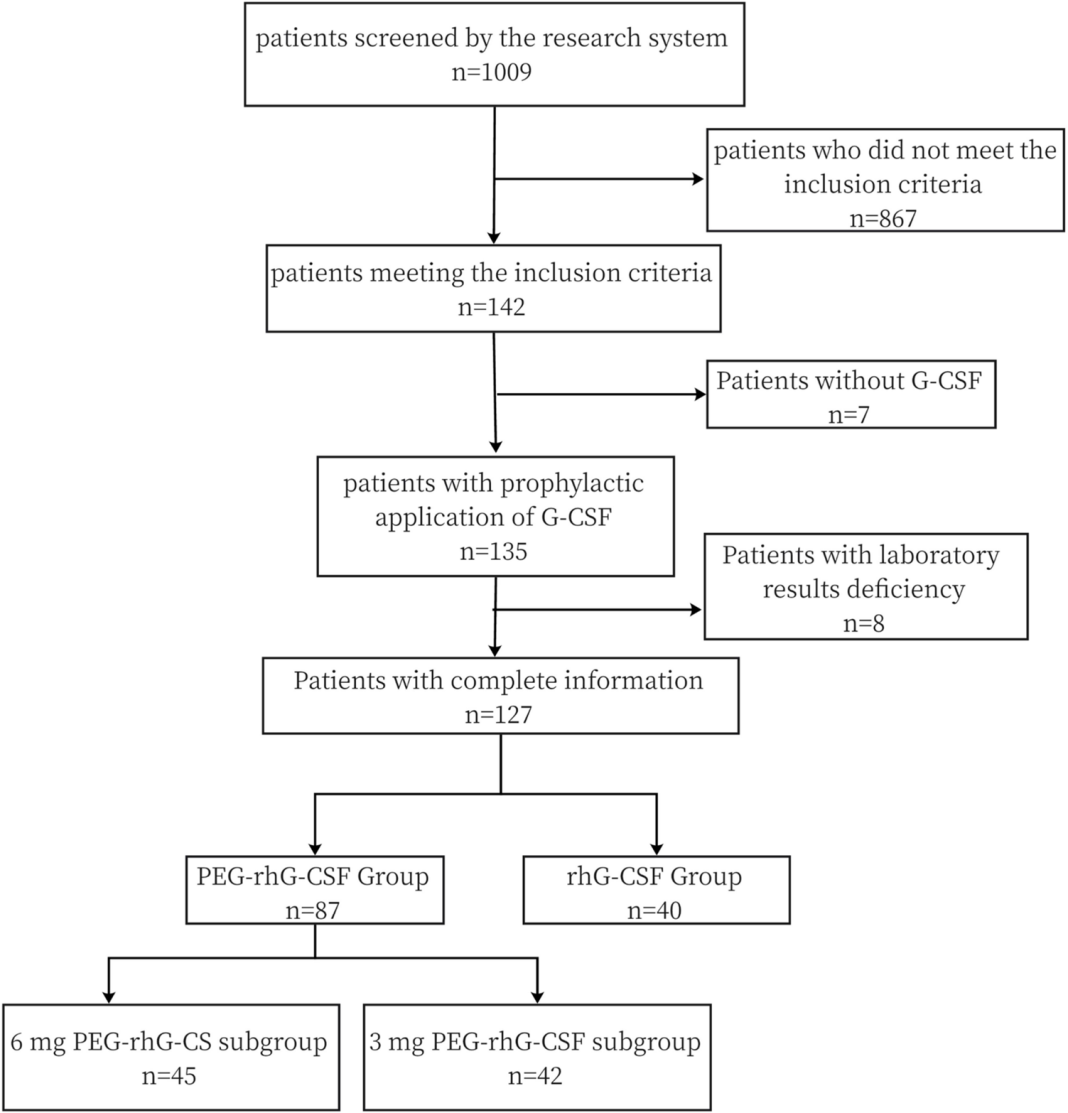
Trial flow chart

As shown in Fig. 1, a total of 127 patients with stage I-III breast cancer were enrolled in this study. All patients had completed at least 2 cycles of EC chemotherapy. 87 patients were divided into trial groups, including 45 patients in the 6 mg PEG-rhG-CSF subgroup and 42 patients in the 3 mg PEG-rhG-CSF subgroup, and 40 patients were divided into rhG-CSF group. The age distribution differed between the two long-acting subgroups, with the mean age of patients in the 6 mg PEG-rhG-CSF subgroup being older than that in the 3 mg subgroup (*p* = 0.025). If the age grouping was based on a cut-off value of 50 years old, 31 patients in the 6 mg PEG-rhG-CSF subgroup were 50 years and older compared to 16 patients in the 3 mg subgroup, a significant difference (*p* = 0.007). There was no statistical difference in the weight distribution of patients between the two long-acting subgroups, with all patients in the 6 mg subgroup weighing over 45 kg and one patient in the 3 mg subgroup weighing < 45 kg. Other clinical characteristics and baseline WBC and ANC levels were not statistically different among the three groups and were comparable, as detailed in Table 1.

**Table 1:** Baseline characteristics of breast cancer patients (Total = 127, n (%))

	PEG-rhG-CSF 6 mg (*N* = 45)	PEG-rhG-CSF 3 mg (*N* = 42)	rhG-CSF (*N* = 40)	F/χ^2^	*P*
Age (years)				2.89	0.059
Mean (± SD)	52.02 (± 8.93)	47.24 (± 11.02)	48.23 (± 9.43)		
Weight (kg)				2.98	0.054
Mean (± SD)	62.28 (± 9.25)	58.81 (± 8.30)	63.81 (± 11.00)		
Family history				0.66	0.719
Yes	8 (17.8)	9 (21.4)	10 (25.0)		
No	37 (82.2)	33 (78.6)	30 (75.0)		
Menstrual status				2.44	0.295
Menopause	23 (51.1)	17 (40.5)	23 (57.5)		
Non Menopause	22 (48.9)	25 (59.5)	17 (42.5)		
TNM stage				3.45	0.489
I	11 (24.4)	7 (16.7)	6 (15.0)		
II	21 (46.7)	26 (61.9)	26 (65.0)		
III	13 (28.9)	9 (21.4)	8 (20.0)		
LN metastasis				6.44	0.376
0	16 (35.6)^c^	13 (31.0)	18 (45.0)		
1 ~ 3	17 (37.8)	19 (45.2)	14 (35.0)		
4 ~ 9	7 (15.6)	7 (16.7)	8 (20.0)		
≥ 10	5 (11.1)	3 (7.1)	0 (0.0)		
Pathological type				-^b^	0.592
Invasive ductal carcinoma	43 (95.6)	40 (95.2)	36 (90.0)		
Other^a^	2 (4.4)	2 (4.8)	4 (10.0)		
Histological grade				-^b^	0.326
I	2 (4.4)^c^	1 (2.4)	5 (12.5)		
II	20 (44.4)	24 (57.1)	19 (47.5)		
III	23 (51.1)	17 (40.5)	16 (40.0)		
ER status				0.311	0.856
negative	18 (40.0)	19 (44.4)	18 (45.0)		
positive	27 (60.0)	23 (54.8)	22 (55.0)		
PR status				3.349	0.187
negative	17 (37.8)	19 (45.2)	23 (57.5)		
positive	28 (62.2)	23 (54.8)	17 (42.5)		
HER-2 status				0.163	0.922
negative	30 (66.7)	27 (64.3)	25 (62.5)		
positive	15 (33.3)	15 (35.7)	15 (37.5)		
Ki67 expression (%)				0.794	0.672
≤ 20	13 (28.9)	11 (26.2)	14 (35.0)		
> 20	32 (71.1)	31 (73.8)	26 (65.0)		
WBC (× 10^9^/L)				1.72	0.183
Mean (± SD)	5.82 (± 1.53)	6.39 (± 1.41)	5.83 (± 1.90)		
ANC (× 10^9^/L)				2.15	0.121
Mean (± SD)	3.58 (± 1.31)	4.12 (± 1.24)	3.59 (± 1.50)		

*Abbreviations: SD* standard deviation, *TNM* Tumor node metastasis, *LN* Lymph node, *HER-2* Human epidermal growth factor receptor-2, *WBC* White blood cell, *ANC* Absolute neutrophil count

^a^Including medullary carcinoma, Neuroendocrine carcinoma, Apocrine carcinoma, and So on, ^b^Adopt Fisher exact test, ^c^The total may not be 100% due to rounding

### Efficacy

The classification of the degree of leukocyte and neutrophil decline in this study was based on CTCAE 5.0, as listed in Table 2.

**Table 2:** Myelosuppression grade

Grade	WBC (× 10^9^/L)	ANC (× 10^9^/L)	HGB (g/L)	PLT (× 10^9^/L)
0	≥ 4.0	≥ 2.0	≥ 110	≥ 100
I	3.0–3.9	1.5–1.9	100–109	75–99
II	2.0–2.9	1.0–1.4	80–99	50–74
III	1.0–1.9	0.5–0.9	60–79	25–49
IV	< 1.0	< 0.5	< 60	< 25

*Abbreviations: WBC* White blood cell, *ANC* Absolute neutrophil count, *HGB* Hemoglobin, *PLT* Platelet

### The primary efficacy endpoint– the incidence of FN in cycle 1

**Fig. 2:**
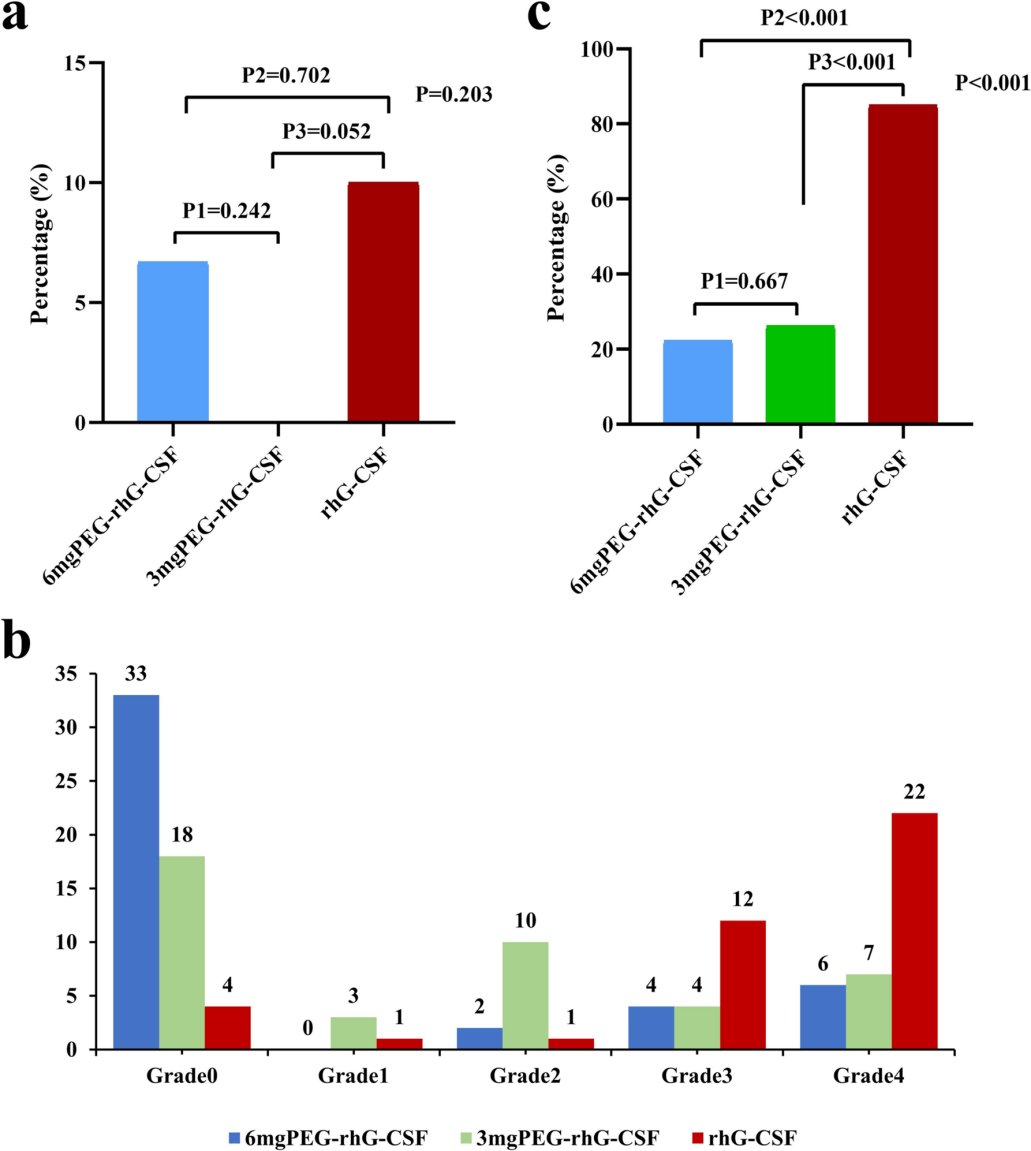
**a** Incidence of FN in cycle 1, **b** Incidence of different grade CIN in the three groups, **c** Incidence of grade ≥ 3 CIN

There was no statistical difference in the incidence of FN in cycle 1 among the three groups (*p* = 0.203), with 6.7% (3/45) of patients in the 6 mg PEG-rhG-CSF subgroup and 10% (4/40) in the rhG-CSF group showing a slightly higher incidence, while none of patients in 3 mg subgroup experienced FN, but none of the comparisons between the two long-acting subgroups were also statistically significant. See Fig. 2a for details.

### The secondary efficacy endpoint

The lowest value in each patient's routine blood data was selected for inclusion in the analysis, and it was found that the proportion and degree of myelosuppression occurred differently among groups. Patients in the two subgroups of PEG-rhG-CSF had a milder degree of CIN, mainly at degree 2 and below, with the incidence of grade ≤ 2 CIN in the 6 mg PEG-rhG-CSF subgroup accounting for 77.8% (35/45), 3 mg PEG-rhG-CSF subgroup accounting for 73.8% (31/42), while the rhG-CSF group occurred with a more severe degree of CIN, predominantly grade ≥ 3. See Fig. 2b for details.

#### *The incidence of grade* ≥ *3 CIN*

As shown in Fig. 2c, the incidence of grade ≥ 3 CIN was significantly lower in both PEG-rhG-CSF subgroups than in the rhG-CSF group, with 22.2% (10/45) having the lowest incidence in 6 mg PEG-rhG-CSF subgroup, followed by 26.2% (11/42) in 3 mg subgroup and 85% (34/40) in the rhG-CSF group, with a statistically significant comparison among the three groups (*P* < 0.001).

There was no significant difference between the two subgroups of PEG-rhG-CSF for this study endpoint, but considering the different age distribution and avoiding confounding the results by age, the results were stratified using an age cut-off of 50 years as shown in Table 3. In patients aged 50 years and younger, the 3 mg subgroup also had a good clinical benefit, with no statistical difference. However, the incidence of grade ≥ 3 CIN in older patients was lower in the 6 mg PEG-rhG-CSF subgroup than (22.6 vs. 56.25%, *p* = 0.021).

**Table 3:** Incidence of grade ≥ 3 CIN under age stratification (n (%))

Age (years)	6 mg PEG-rhG-CSF	3 mg PEG-rhG-CSF	P
≤ 50			0.337
Yes	3 (21.4)	2 (8.3)	
No	11 (78.6)	22 (91.7)	
> 50			0.021
Yes	7 (22.6)	9 (56.25)	
No	24 (77.4)	7 (43.75)	

#### Distribution of WBC and ANC

**Fig. 3:**
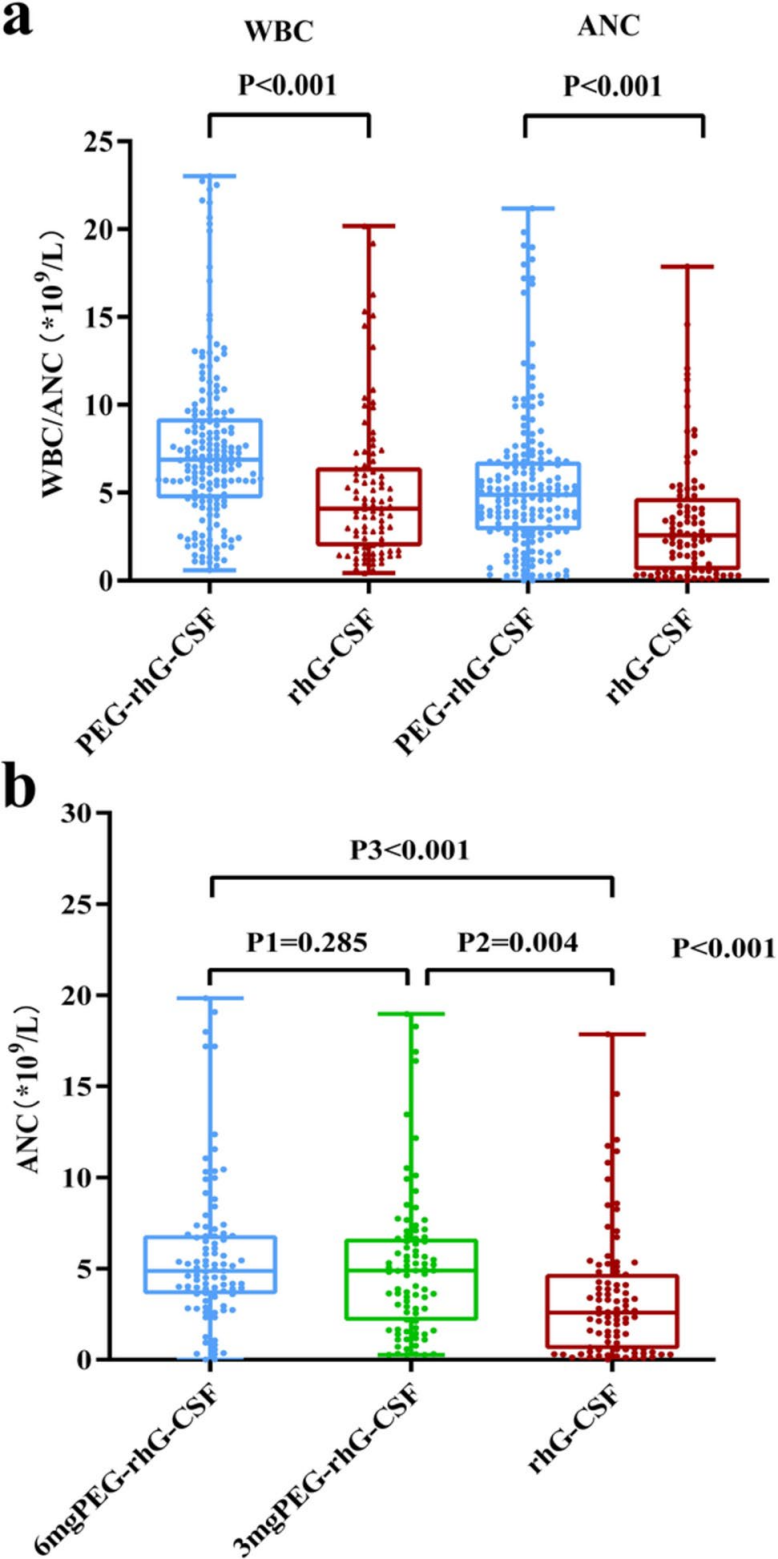
**a** The overall levels of WBC and ANC, **b** Comparison of median ANC for the three groups

A total of 274 routine blood tests were collected after cycle 1. After excluding one extreme value, 93, 88, and 90 routine blood data were retained in the 6 mg, 3 mg PEG-rhG-CSF subgroup, and rhG-CSF group respectively. The overall distribution of WBC and ANC levels in the long-acting group was significantly higher than those in short-acting group, with statistical differences (Fig. 3a). Advantages were obtained for each dose long-acting subgroup compared with the rhG-CSF group, but there was no significant difference between the two long-acting subgroups, where the median ANC in the 6 mg subgroup was 4.88 (95% CI: 4.88 ~ 6.54) × 10^9^/L and 3 mg subgroup was 4.89 (95% CI: 4.26 ~ 5.93) × 10^9^/L, while the rhG-CSF group had a median of 2.56 (95% CI: 2.67 ~ 4.12) × 10^9^/L, which was significantly lower than the two long-acting subgroups mentioned above (Fig. 3b).

#### The nadir values of ANC

As shown in Table 4, the nadir values of ANC differed among the three groups significantly, with the highest in the 6 mg PEG-rhG-CSF subgroup and the lowest in the rhG-CSF group (*p* < 0.001). There was no significant difference between the two long-acting subgroups.

**Table 4:** The nadir value of ANC in cycle 1 among the three groups

	nadir value of ANC (× 10^9^/L) Mean (± SD)	P
6 mg PEG-rhG-CSF vs. rhG-CSF	3.90 ± 2.15 vs. 2.07 ± 2.66	0.001
3 mg PEG-rhG-CSF vs. rhG-CSF	3.06 ± 2.24 vs. 2.07 ± 2.66	0.059
6 mg vs. 3 mg PEG-rhG-CSF	3.90 ± 2.15 vs. 3.06 ± 2.24	0.101
Three groups	3.90 ± 2.15 vs. 3.06 ± 2.24 vs. 2.07 ± 2.66	0.002

#### Comparison of WBC and ANC curves over time

**Fig. 4:**
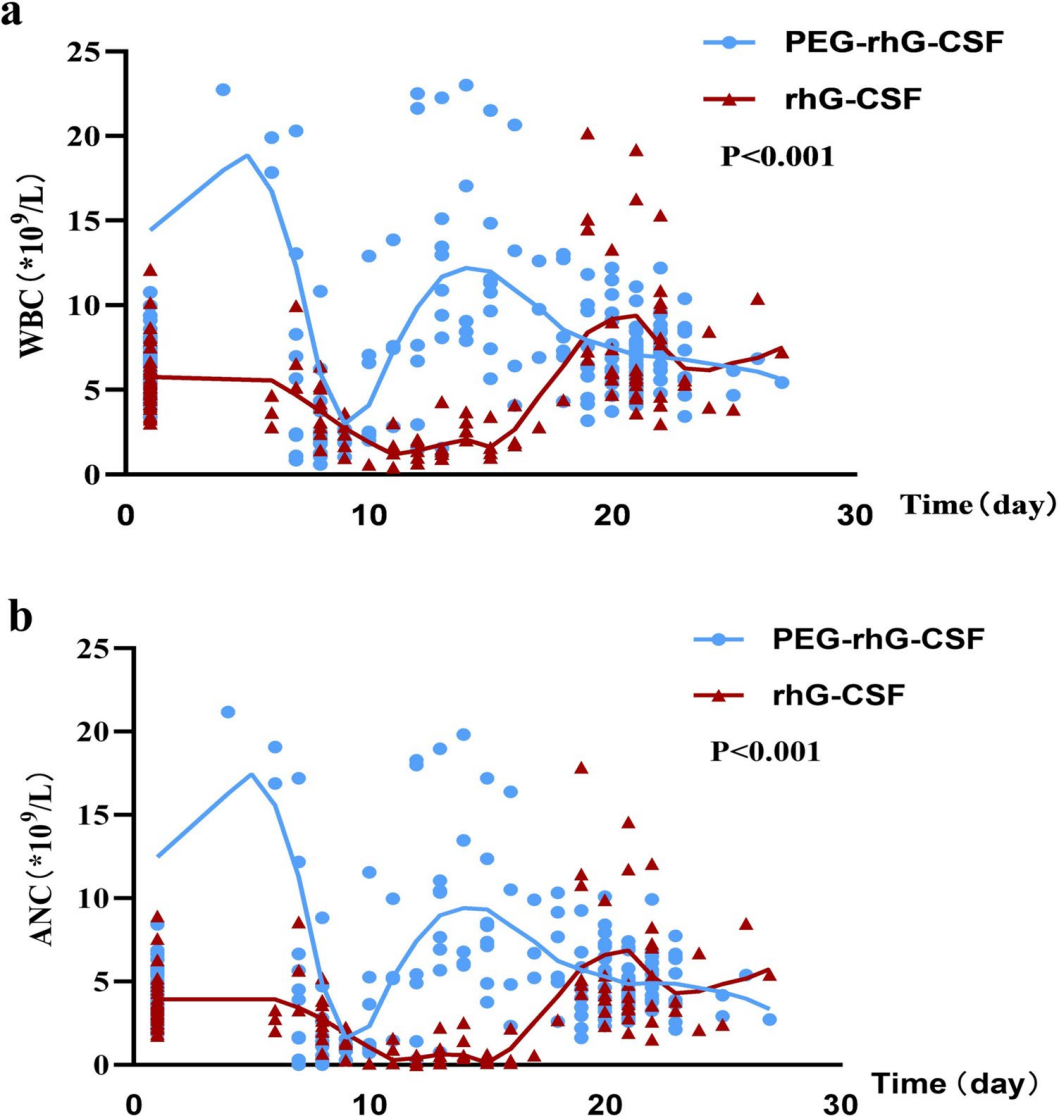
**a** Comparison of WBC curves over time, **b** Comparison of ANC curves over time

The routine blood data collected in cycle 1 were labeled according to the time of collection, and the fitted curves of WBC and ANC over time between the two major groups of long- and short-acting G-CSF were plotted separately. As shown in Fig. 4a and b, the WBC and ANC in the long-acting group started to drop around day 6 after treatment, and dropped to the lowest point on day 9–10 and rebounded rapidly, with a short myelosuppression period; the short-acting group started to drop at the same time and dropped to the lowest point, but the curve of this group showed that the myelosuppression period lasted from day 10 to around day 14, and rebounded to the same level as the long-acting group around day 20.

#### The incidence of CIN-related complications

**Fig. 5:**
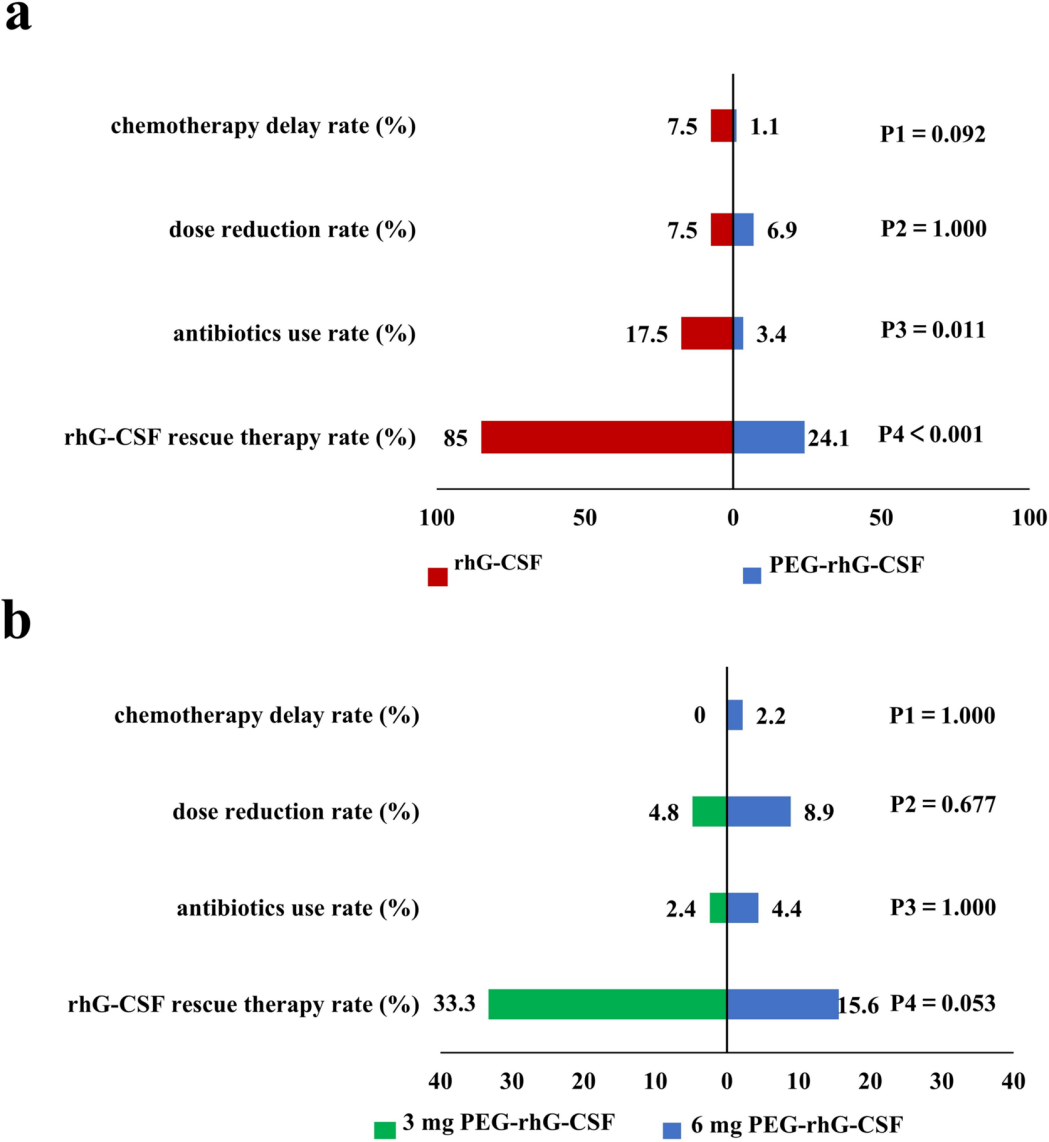
**a** Comparison of CIN-related complications between PEG-rhG-CSF and rhG-CSF, **b** Comparison of CIN-related complications between the two PEG-rhG-CSF subgroups.

As shown in Fig. 5a, one patient (1.1%) in the PEG-rhG-CSF group had a 3-day delay in chemotherapy due to CIN, while 3 patients (7.5%) in the rhG-CSF group had a 3 ~ 6 days delay in chemotherapy. There was no statistical difference at *P* = 0.092. Dose reduction rates were similar in both groups (6.9 vs. 7.5%), but the long-acting group had a lower rate of antibiotic use (3.4 vs. 17.5%, *P* = 0.011) and rhG-CSF rescue therapy (24.1 vs. 85.0%, *P* < 0.001).

As shown in Fig. 5b, the two long-acting subgroups were essentially similar in terms of chemotherapy delay, dose reduction, and antibiotic use. Although there was no statistical difference between the two groups in any of CIN-related complications including the rhG-CSF rescue treatment rate (*P* > 0.05), the higher rhG-CSF rescue treatment rate in the 3 mg subgroup, approximately twice that of the 6 mg subgroup (33.3 vs. 15.6%), was still clinically significant.

### Hematologic adverse events

There were no statistical differences in hematological and non-hematological adverse reactions among the groups, as detailed in Table 5. The non-hematological adverse reactions in all groups were mainly bone pain and gastrointestinal reactions. Except for two cases of grade 3 nausea and one case of grade 3 liver injury in the 3 mg PEG-rhG-CSF subgroup and one case of grade 3 anemia in the rhG-CSF group, all other adverse reactions were of grade 1 ~ 2 and well tolerated by patients.

**Table 5:** Adverse events in three groups (n (%))

AEs	6 mg PEG-rhG-CSF	3 mg PEG-rhG-CSF	rhG-CSF	P
Hematology AEs				
Anemia	4 (8.9)	4 (9.5)	9 (22.5)	> 0.05
Thrombocytopenia	3 (6.7)	3 (7.1)	3 (7.5)	> 0.05
Leukocytosis	17 (37.8)	12 (28.6)	10 (25.0)	> 0.05
Neutrophilia	14 (31.1)	12 (28.6)	10 (25.0)	> 0.05
Non-hematology AEs				
Bone pain	2 (4.4)	2 (4.8)	1 (2.5)	> 0.05
Nausea	5 (11.1)	5 (11.9)	7 (17.5)	> 0.05
Anorexia	5 (11.)	2 (4.8)	2 (5.0)	> 0.05
Fatigue	1 (2.2)	1 (2.4)	2 (5.0)	> 0.05
Rash	1 (2.2)	0 (0.0)	0 (0.0)	> 0.05
Liver injury	1 (2.2)	1 (2.4)	1 (2.5)	> 0.05

### The timing of rhG-CSF rescue treatment

A total of 21 patients in the PEG-rhG-CSF group were treated with rhG-CSF rescue therapy, including 1, 4, 3, and 13 patients with grade 1 ~ 4 CIN each. It was found that the patients with grade 1 CIN had developed febrile infections, most patients with grade 2 CIN had not passed the minimum point of myelosuppression at the time of routine blood tests, and two patients with grade 3 CIN had myelosuppression for more than 7 days. A total of 34 patients in the rhG-CSF group underwent rhG-CSF rescue therapy, including 22 patients with grade 4 CIN, 11 with grade 3 and 1 with grade 2 CIN. The patient with grade 2 CIN was treated with rhG-CSF and antibiotics as he developed a fever, cough, and other symptoms of lung infection after chemotherapy. In summary, the majority of patients treated for CIN were in grade 4 (63.6%) and grade 3 (25.5%), with some patients in grade 1 ~ 2 who were at high risk of infection or had developed an infection.

## Discussion

Chemotherapy plays an important role in the systemic treatment of breast cancer, and the reduction of chemotherapy toxicity, especially hematological dose-limiting toxicity, has been a major focus of research by oncologists. The efficacy and safety of the two types of G-CSF have been the subject of much research, but there is still no consensus, particularly in breast cancer patients [13, 20]. In our study, although our sample size was not large, all our patients were treated with the EC regimen (Epirubicin: 90 mg/m^2^ and Cyclophosphamide: 600 mg/m^2^), and the treatment interval and medication giving modalities were uniform. It is more reliable to observe the incidence of FN and CIN in each group of patients under the same treatment throughout the study. Such a uniform treatment regimen is actually rare in the articles observing the incidence of FN and CIN, so this is our advantage.

The main purpose of G-CSF in clinical practice is to reduce the incidence of infections with FN as the main manifestation. A study by Xie J et al. found that the incidence of FN was only observed in cycle 1 in both the PEG-rhG-CSF and rhG-CSF groups, with no statistical difference in efficacy between the two groups [26]. However, some studies have reported that prophylactic application of PEG-rhG-CSF reduced the incidence of FN by 73% and rhG-CSF by 57%, with a significant difference in efficacy [21]. The differences in clinical characteristics of patients, chemotherapy regimens and doses administered, and the use of prophylactic antibiotics in different studies may also account for the differences in the conclusions of the studies.

The results of our study showed that the incidence of FN in cycle 1 was lower in the PEG-rhG-CSF group, but the difference was not statistically significant (*P* = 0.203), while a significant advantage was obtained in both dose subgroups of PEG-rhG-CSF in reducing the incidence of grade ≥ 3 CIN (22.2 vs. 26.2 vs. 85%, *P* < 0.001). This is consistent with the findings of Wang WB et al., which included all breast cancer patients, where PEG-rhG-CSF also gained a statistical advantage in the incidence of grade ≥ 3 CIN (*P* = 0.006) [27]. Therefore, considering the results of this study and the Meta-analysis above, the benefit of primary prophylaxis with PEG-rhG-CSF may be more pronounced in breast cancer patients. In addition, the Chinese Expert Consensus on the Clinical Use of PEG-rhG-CSF (2016 version) states that PEG-rhG-CSF has a stronger and longer-lasting effect in elevating ANC [12]. This was confirmed by the fact that the overall WBC and ANC levels and the lowest ANC value were significantly higher in the PEG-rhG-CSF group (*P* = 0.002), and the duration of myelosuppression was shorter in this group.

Severe CIN and FN not only add to the clinical burden of the patient but also the financial burden [28, 29]. Therefore, the incidence of CIN-related complications is also an important indicator to assess the efficacy of G-CSF. The study of He Jingjing demonstrated that the application of PEG-rhG-CSF for primary prophylaxis resulted in lower rates of chemotherapy delay (13.3 vs. 16.6%, *P *< 0.05) and dose reduction (13.3 vs. 20.0%, *P* > 0.05) compared to rhG-CSF [24]. In our study, PEG-rhG-CSF group also showed lower rates of CIN-related complications than the rhG-CSF group, with statistically significant differences in both antibiotic use rate (4 vs. 17.5%, *P* = 0.011) and rhG-CSF rescue treatment rate (24.1 vs. 85.0%, *P* < 0.001). The delayed chemotherapy rate (1.1 vs. 7.5%, *P* > 0.05) and dose reduction rate (6.9 vs. 7.5%, *P* > 0.05) were also lower in the PEG-rhG-CSF group, but the data for these two indicators were lower in our study compared to previous reports. Considering that, on the one hand, this was related to the lack of rigor in the implementation of the retrospective study protocol, with three and two patients in the PEG-rhG-CSF and rhG-CSF groups respectively delayed therapy due to personal reasons. On the other hand, as this study was a single-center study with a small sample size, the influence of selection bias on the results could not be excluded.

PEG-rhG-CSF is mainly administered at a fixed dose of 6 mg, but studies have demonstrated that this dose causes leukocytosis in patients and may cause excessive depletion of bone marrow when administering dose-intensive chemotherapy [22]. In addition, as studies have progressed, it has been demonstrated that patients are at increased risk of secondary malignancies such as acute myeloid leukemia and myelodysplastic syndromes following G-CSF administration [30]. In light of these potential toxicities and the era of precision medicine, more and more scholars are reexamining the dose of PEG-rhG-CSF and have conducted a series of studies. A Japanese phase II dose creep study in breast cancer patients showed that the efficacy of PEG-rhG-CSF was positively correlated with dose, but reached its peak efficacy at 3.6 mg [31]. A multicenter, single-arm, phase II clinical study by Qi Mei et al. enrolled 151 patients of all cancer types with intermediate-risk chemotherapy for FN and ≥ 1 combined risk factors for half-dose PEG-rhG-CSF prophylaxis and found good results in reducing the incidence of FN (3.3%), delayed chemotherapy (4.0%), antibiotic use (15.2%), chemotherapy interruption (9.3%) and adverse events rate of 18.5%, which was well tolerated by patients [32]. Studies exploring the prophylactic effect of half-dose PEG-rhG-CSF have also demonstrated clinical benefits in some chemotherapy regimens [7, 33]. Our study compared the efficacy and tolerability of PEG-rhG-CSF at doses of 6 mg and 3 mg in breast cancer patients treated with EC adjuvant chemotherapy and showed that the incidence of cycle 1 FN (6.7 vs. 0%), grade ≥ 3 CIN (22.2 vs. 26.2%), ANC nadir (3.90 ± 2.15 vs. 3.06 ± 2.24) × 10^9^/L, CIN-related complications and the incidence of adverse events were not statistically different. However, it was worth noting that the incidence of grade ≥ 3 CIN was significantly lower in the 6 mg subgroup (22.6 vs. 56.25%, *P* = 0.021) in the 50 + age group, considering that the 6 mg dose PEG-rhG-CSF may be of more benefit in older patients with poor bone marrow conditions. In addition, as seen in Fig. 3b, although the distribution of ANC levels was not statistically different between the two subgroups, patients in the 6 mg subgroup had a more concentrated distribution, whereas the 3 mg subgroup had a greater degree of dispersion. So it cannot be denied that although there was a clinical benefit in the 3 mg subgroup, the 6 mg dose administered had a smoother and less fluctuating effect, and therefore the patient's FN risk needs to be carefully assessed when selecting the dose in practice. Whether there is a broader benefit from half-dose PEG-rhG-CSF needs to be studied in different cancer types, different chemotherapy regimens, and even different ethnic groups, taking into account the patient's chemotherapy regimen, age, bone marrow function, and economic situation.

Some adverse drug reactions are inevitable with G-CSF, but most are mild and tolerable. In a recent Meta analysis of adverse events about G-CSF showed that the occurrence of adverse events were common, with musculoskeletal aches and pains and gastrointestinal reactions predominating [34]. From initial registration studies during the development phase of PEG-rhG-CSF, which demonstrated that both were well tolerated, to later comparative studies in the real world, which showed a general agreement in terms of the incidence of adverse reactions [3, 17]. In our study, no rare adverse reactions were observed in any of the patients, with leukocytosis (25 ~ 37.8%) being the predominant hematological adverse reaction and non-hematological adverse reactions such as nausea and anorexia (22.5 ~ 26.7%) and bone pain (2.5 ~ 4.4%), mostly of grade 1 ~ 2. There was no statistical difference among the groups, which was consistent with previous reports. The incidence of bone pain recorded in our study was similar to the study conducted by Professor Li HP et al. (3.7%) [10]. In addition, our study found that the 3 mg subgroup did not significantly reduce the incidence of leukocytosis compared to the 6 mg subgroup, which is consistent with the findings of Cao Wei et al. [22].

Prophylactic application of G-CSF does not achieve 100% prophylaxis, which requires clinicians to be aware of the timing of rhG-CSF rescue therapy. CSCO guidelines recommend considering rhG-CSF rescue therapy in patients with prophylactic PEG-rhG-CSF if the ANC is < 0.5 × 10^9^/L and lasts ≥ 3 days [5]. However, some studies have reported that only a few physicians are clear about the timing of rhG-CSF therapeutic application, and it has been suggested that this phenomenon is partly due to the lack of awareness of the dangers of severe CIN and FN among physicians [35]. Few studies have been reported on the timing of real-world rhG-CSF rescue therapy in China. In view of the above situation, this study analyzed the clinical data of patients receiving rhG-CSF rescue therapy. The results showed that the majority of patients receiving rhG-CSF rescue therapy were with grade 4 CIN (63.6%) and grade 3 CIN (25.5%) and 10.9% with grade 1 ~ 2 CIN. The majority of patients with grade 1 ~ 2 CIN were found to be at high risk of infection or already co-infection. In addition, some patients with grade 4 CIN and 3 days of duration were not given rhG-CSF rescue therapy according to the guidelines in the study. Although no serious cases such as infection were recorded, this does not exclude the distortion of results due to sample size limitation.

This was a single-center retrospective study and longitudinal comparisons from cycles 1 ~ 4 are limited by the fact that some patients changed prophylactic drugs in cycle 2, making it impossible to maintain a uniform study population. Previous studies have shown that the difference in efficacy between the two G-CSF differs from cycles 1 ~ 4 and that the benefit of PEG-rhG-CSF may be more pronounced in multiple cycles of chemotherapy, although the exact mechanism is unclear [26]. Further prospective studies could be conducted to compare the efficacy and explore the mechanisms involved in multiple cycles of chemotherapy.

To sum up, there are some limitations to this study. Firstly, due to the limited sample size, some study results may be slightly biased. The application of the results needs to be combined with studies with larger sample sizes. Secondly, this study was a retrospective study, which was unable to obtain the blood routine data of all patients with close and consistent detection time like clinical trials, so there may be a low incidence of outcome indicators due to missed detection.

In addition, we find that, in clinical practice, the course of use of rhG-CSF is almost shorter than the course recommended by the guidelines, due to the economic conditions of patients, the inconvenient return to hospital, the shortage of medical resources, the COVID-19 epidemic and other factors. Moreover, for patients receiving one- or two-week intensive chemotherapy, the prophylactic application of a long course of rhG-CSF may cause excessive bone marrow consumption. Therefore, the phenomenon of insufficient treatment course of rhG-CSF is very common, and most patients will stop the drug before the prescribed course of treatment. In the same way, in this study, the duration of prophylactic application of rhG-CSF was 3–5 days, which was shorter than the recommended course of use in the guidelines. While, in fact, this study also proves that the purpose may be achieved without a particularly long course of treatment, and the application of G-CSF will cause bone pain, considering the feeling of patients, as long as the efficacy can be guaranteed, it may not need to continue to inject for too long time, which may become a choice in clinical work.

As this study was conducted on a single cancer type and chemotherapy regimen, the applicability of the findings to other cancer types and chemotherapy regimens needs to be further clarified in a multi-cancer, large sample size study. The optimal dose of PEG-rhG-CSF can be further explored in different cancer types and different chemotherapy regimens to minimize the incidence of CIN and FN and the potential harm of G-CSF.

## Conclusion

Compared with rhG-CSF, the application of PEG-rhG-CSF for primary prevention is more advantageous in reducing myelosuppression of chemotherapy and CIN-related complications in breast cancer patients, and both are similarly tolerated, which has high clinical application value. PEG-rhG-CSF at 3 mg half dose has also shown good efficacy in preventing CIN, but its usage in clinical practice requires a comprehensive assessment of FN risk and economic situation in relation to the patient's chemotherapy regimen, age, and bone marrow function. Consider rhG-CSF rescue or even antibiotic therapy if grade ≥ 3 CIN develops after prophylactic G-CSF and in other patients assessed to be at high risk of infection or already co-infected.

## Data Availability

The original contributions presented in the study are included in the article/Supplementary Material, further inquiries can be directed to the corresponding author.

## Abbreviations

ANC: Absolute neutrophil count
ASCO: American Society of Clinical Oncology
AEs: Adverse events
CIN: Chemotherapy-induced-neutropenia
CTCAE: Common Terminology Criteria for Adverse Events
CSCO: Chinese Society of Clinical Oncology
CIs: Confidence Intervals
EC: Epirubicin and cyclophosphamide
ECOG: Eastern Cooperative Oncology Group
FN: Febrile neutropenia
G-CSF: Granulocyte colony-stimulating factor
HR: Hormone receptor
HER-2: Human epidermal growth factor receptor-2
HGB: Hemoglobin
LN: Lymph node
NCCN: National comprehensive cancer network
PEG-rhG-CSF: Pegylated recombinant human granulocyte colony-stimulating factor
PLT: Platelet
RhG-CSF: Recombinant human granulocyte colony-stimulating factor
SD: Standard deviation
TNM: Tumor node metastasis
WBC: White blood cells
